# A Conserved Gene Structure and Expression Regulation of miR-433 and miR-127 in Mammals

**DOI:** 10.1371/journal.pone.0007829

**Published:** 2009-11-25

**Authors:** Guisheng Song, Li Wang

**Affiliations:** Departments of Medicine and Oncological Sciences, Huntsman Cancer Institute, University of Utah School of Medicine, Salt Lake City, Utah, United States of America; Institute of Genetics and Molecular and Cellular Biology, France

## Abstract

MicroRNAs play essential roles in many cellular processes. However, limited information is available regarding the gene structure and transcriptional regulation of miRNAs. We explored the gene cluster encoding miR-433/127 in mammalian species using bioinformatics and *in vitro* “gene” expression approaches. Multiple sequence alignments (MSA) showed that the precursors of miR-433 and of miR-127 exhibited 95% and 100% similarity, respectively, in human, chimpanzee, horse, dog, monkey, rat, cow, and mouse. MSA of the promoter sequences of miR-433 and of miR-127 revealed lower sequence similarity among these mammalian species. However, the distance between miR-433 and miR-127 was strikingly similar, which was between 986 and 1007 bp and the position of transcription factor (TF) binding motifs, including estrogen related receptor response element (ERRE), was well conserved. Transient transfection assays showed that promoters of miR-433 and of miR-127 from human, rat, and dog were activated by estrogen related receptor gamma (ERRγ) and inhibited by small heterodimer partner (SHP). ChIP assays confirmed the physical association of ERRγ with the endogenous promoters of miR-433 and miR-127. *In vitro* over-expression of the human, rat, or dog miR-433/127 loci in cells, using an expression vector containing miR-433/127 and their promoter regions, markedly induced a differential expression of both primary and mature miR-433 and miR-127, indicating that miR-433 and miR-127 were possessed from their independent promoters. Our studies for the first time demonstrate a conserved gene structure and transcriptional regulation of miR-433 and miR-127 in mammals. The data suggest that the miR-433/127 loci may have evolved from a common gene of origin.

## Introduction

MicroRNAs (miRNAs, miRs) is a class of short noncoding RNA molecules that post-transcriptionally regulate gene expression by targeting mRNA, leading to translational repression or degradation of the target mRNA [1]. Despite the growing evidence for their importance in development, metabolism and carcinogenesis [2], limited information is available about the transcriptional regulation of miRNA expression. The future challenges are to determine how miRNAs are regulated transcriptionally, to identify the biological targets of miRNAs, and the signaling pathways they regulated under various physiological and pathological conditions.

To determine the transcriptional control of miRNAs, we set up a computational and database mining approach. This resulted in the cloning of the gene cluster encoding mouse miR-433 and miR-127, which provided the first report for an overlapping code usage of the paired miR-433/127 gene [3]. Based on their overlapping gene structure and transcriptional initiation and termination sites, we subsequently cloned promoters of miR-433 and miR-127. We elucidated a common regulatory mechanism governing miR-433 and miR-127 promoter activities, which was dependent on nuclear receptor estrogen related receptor gamma (ERRγ, NR3B3) and small heterodimer partner (*SHP*, NR0B2) [4]. However, the question remains to be determined whether molecular details of miR-433 and miR-127 regulation by ERR/SHP are restricted to mouse or whether they apply to other species.

In the present study, we analyzed genes encoding miR-433 and miR-127 and determined the promoter transactivation of miR-433 and miR-127 from other mammalian species, including humans. Our studies provide evidence for a conserved gene structure and ERR/SHP dependent regulation of miR-433 and miR-127 gene expression in mammals. Our study suggests that the clustered miRNAs may not usually derive from a single large primary transcript. The data suggest that similar conservation of transcriptional regulation of miRNA expression may occur for other clustered miRNAs in mammalian species.

## Results

### Comparison of Gene Structure of miR-433/127 Loci from Different Mammalian Species

**Figure 1:**
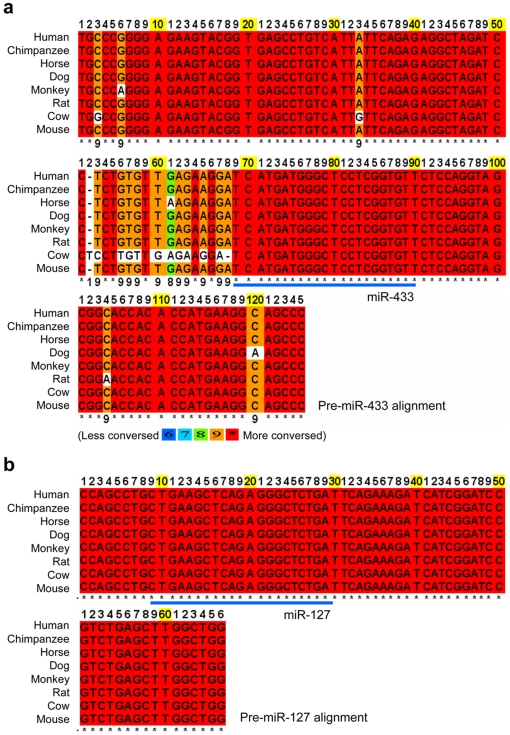
Multiple sequence alignment (MSA) of miR-433 and miR-127 precursor hairpin sequences in eight mammalian species. (**a**) Pre-miR-433 alignment in eight different species. (**b**) Pre-miR-127 alignment in eight different species. ClustalW program was used. Dots indicate nucleotide identify. Nonsynonymous substitutions are indicated with different colors.

We recently have reported that the full length primary transcripts of mouse miR-433 and miR-127 overlapped in a 5′–3′ unidirectional way [3]. An interesting question that we ask is whether a similar gene structure exists in other species, including humans. Using mouse miR-433 and miR-127 precursor hairpin structure sequences as a query, we searched the genome databases of seven other species, including human, chimpanzee, horse, dog, monkey, rat, and cow. The 4.5 kb genomic sequences centered miR-433 and miR-127 were extracted. Although the miR-433/127 loci were located on different chromosomes (Chr) in those species (human, Chr 14; Chimpanzee, Chr14; Horse, Chr 24; Dog, Chr 8; Monkey, Chr 7; Rat, Chr 6; Cow, Chr 21; mouse, Chr 12), multiple sequence alignment (MSA) of the precursors, pre-miR-433 and pre-miR-127, showed that the sequence similarity of pre-miR-433 hairpins was ∼95% (Figure 1a) and of pre-miR-127 was 100% (Figure 1b) among those species. The mature sequences of miR-433 and miR-127 were identical among the eight species.

We next compared the distance between miR-433 and miR-127 precursor hairpin sequences in those eight species. The distance between miR-433 and miR-127 showed a striking similarity: 986 bp in human, chimpanzee, horse, dog, and monkey, 989 bp in rat, 988 bp in cow, and 1007 bp in mouse. The conservation of pre-miR-433 and pre-miR-127 hairpin sequences as well as the distance between them among different mammalian species raised the possibility that miR-433 and miR-127 might be evolved from the same DNA origin during evolution.

### Conserved Transcription Factor Binding Motifs in the Upstream Region of miR-433 and of miR-127 among Mammalian Species

**Figure 2:**
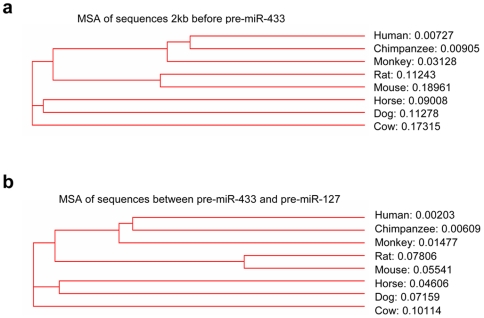
MSA of miR-433 and miR-127 gene promoters in eight mammalian species. **(a)** MSA of sequences 2 kb before pre-miR-433. (**b**) MSA of sequences between pre-miR-433 and pre-miR-127. ClustalW was used to build the phylogenetic tree. The number following each species name represents the evolutionary distance.

DNA sequence alignment is a prerequisite to the identification of virtually all conserved sequence motifs and estimation of evolutionary divergence between sequences. The sequence similarity in either the miR-433 promoter region (Figure 2a) or the genomic region between pre-miR-433 and pre-miR-127 (Figure 2b) was low among the eight species. The genomic region between the two pre-miRNAs is predicted to function as the promoter of miR-127 based on our published mouse data [4]. The lowest evolutionary distance between cow and other species is 0.10114 and the sequence homology is low, based on the sequence alignment of the miR-127 promoter region (Figure 2b).

**Figure 3:**
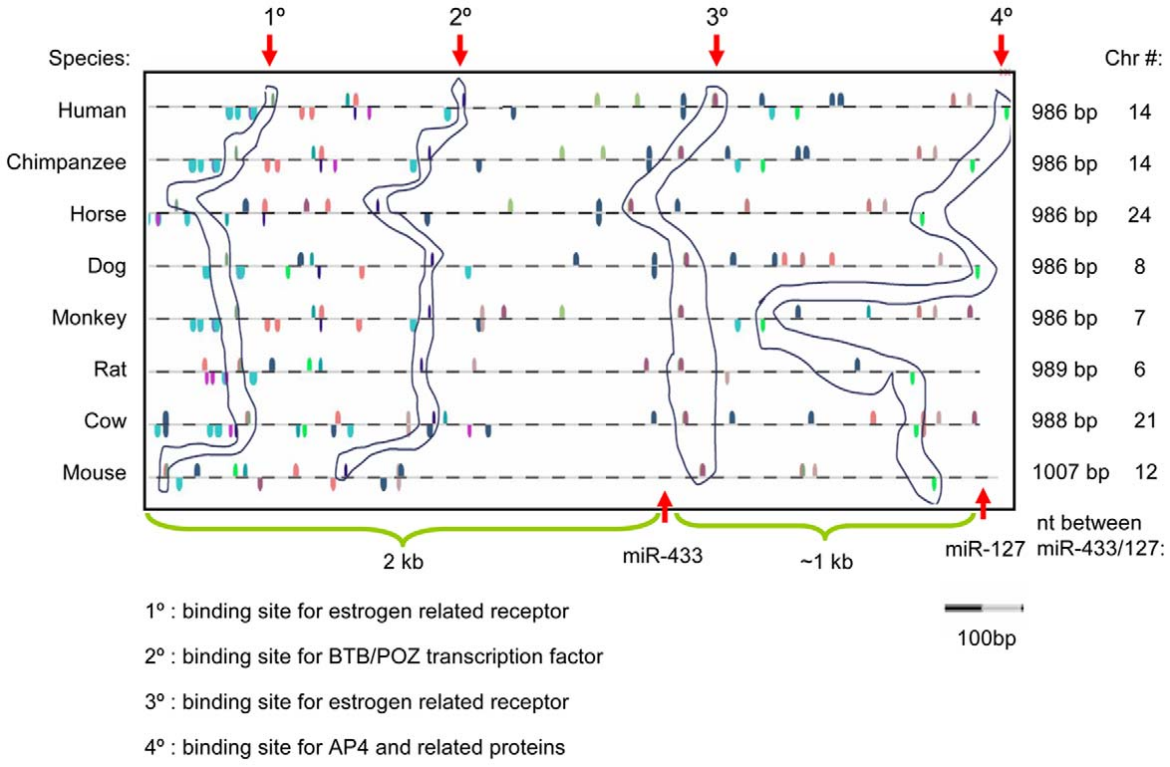
Conserved response elements in the promoters of miR-433 and miR-127 of eight mammalian species. The response elements of the miR-433 and miR-127 genes from eight different species are illustrated. Spots indicated by different colors represent putative binding sites for different transcription factors (TF). Although the miR-433/127 gene loci was located on different chromosomes in different species, the distance between miR-433 and miR-127 is very similar, which is ∼1 kb. Representative common TF binding motifs (1°∼4°) are shown, and their positions appear to be conserved in the promoter region of miR-433 and of miR-127 among different species.

Transcription factor binding sites located in the upstream of miRNA precursor are involved in the expression regulation of miRNAs. We used MatInspector of Genomatix Software Suite to identify transcription factor binding motifs and position preference in the upstream promoter regions of miR-433 and of miR-127 in eight mammalian species. Despite lower sequence similarity, analysis of miR-433 and miR-127 promoters of those species predicted common nuclear receptor binding sites, including ERRE (Figure 3 and Table 1). Interestingly, the position of the common response elements appeared to be conserved among species. Unique potential binding motifs were also identified in the promoter region of miR-433 and of miR-127 in each species. We recently have shown that gene expression of miR-433 and miR-127 in mice was regulated via a nuclear receptor ERRγ/SHP dependent mechanism [4]. Based on the identification of the same binding motifs, we predicted that a common regulatory mechanism of miR-433 and miR-127 expression may exist among different mammalian species.

**Table 1:** Predicted ERRE sites on the miR-433 and miR-127 gene promoters.

Species	ERRE site in miR-promoter region		Nucleotide location (distance between ERRE site and miR-precursor	
	miR-422 pro.	miR-127 pro.	pre-miR-433	pre-miR-127
Human	CGAGGTCA	TGACGTT	1587	577
Rat	CAAGGTCA	TGAGCTT	1531	560
Dog	TGGCCTTG	GTGGTCA	1577	563
Mouse	CAAGTTCA	CAAGGTCA	1533	663

Consensus ERRE: TCAAGGTCA or TGACCTTGA (underline: core sequences).

### Common Regulation of miR-433 and miR-127 Promoter Activity among Mammalian Species

**Figure 4:**
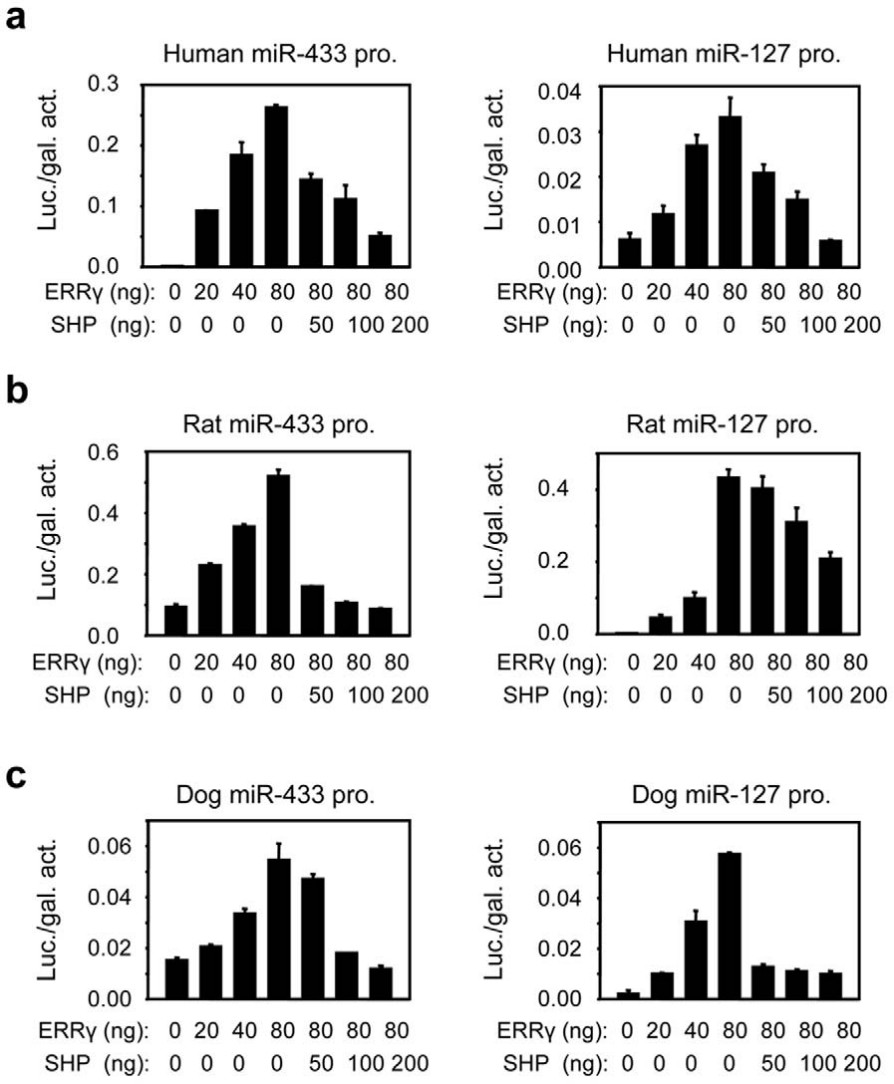
Promoter analysis of miR-433 and miR-127 luciferase reporters of human, rat and dog. Transient transfection assays to determine ERRγ and SHP regulation of miR-433 and miR-127 promoter (pro.) transactivation of human (**a**), rat (**b**), and dog (**c**), respectively. The promoter of pri-miR-433 and of pri-miR-127 from each species was cloned into a pGL3-basic vector, respectively. Hela cells were transfected with the miR-433Luc or miR-127Luc in the presence of nuclear receptor expression plasmids. Luciferase (luc.) activities (act.) were determined, which were normalized by β-gal activities. Data are represented as mean ± SEM.

Based on the above gene structure analysis and sequence prediction referenced from our published mouse data [3], [4], we hypothesized that the genomic location of promoters of miR-433 and miR-127 was similar in other mammalian species as in mouse. We focused on analyzing several representative species, including human, rat, and dog. We cloned the promoters of miR-433 and miR-127 into pGL3 luciferase reporters using genomic DNAs isolated from liver specimens of human, rat and dog. Transient transfection assays were used to elucidate their transcriptional regulation. As expected, ERRγ dose-dependently activated promoters of miR-433 and miR-127 of human (Figure 4a), rat (Figure 4b), and dog (Figure 4c), which was repressed by co-expression of SHP. We also determined the transactivity of two other ERR family members ERRa and ERRβ. ERRa, but not ERRβ, showed strong activation of miR-433 and miR-127 promoters of human, rat, and dog (Figure S1). The results are consistent with the report that ERRγ and ERRa preferentially bind to a similar ERRE motif [5].

**Figure 5:**
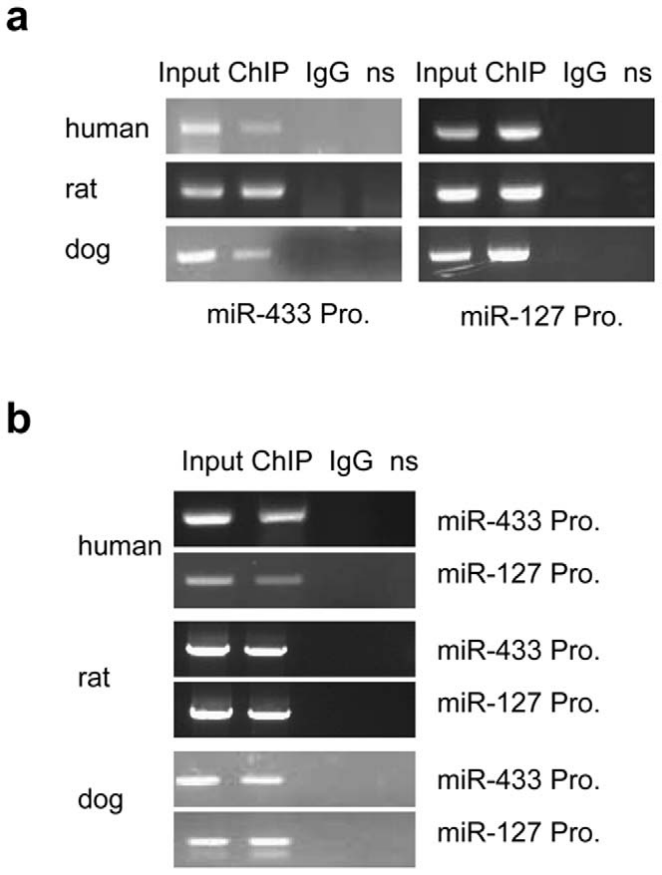
ChIP analysis of ERRγ Co-immunoprecipitation (Co-IP) on the miR-433 and miR-127 promoter region containing putative ERRE in human, rat, and dog. (**a**) The miR-433/127 loci expression plasmid of human, rat, or dog was expressed in mouse Hepa-1 cells (different species), and the binding of ERRγ to the endogenous promoter of miR-433 and of miR-127 of each species was detected using specific ERRγ antibodies. n.s., non-specific, primers ∼10 kb downstream of miR-433 and miR-127 promoter was used, which served as a negative control. (**b**) *In vivo* ChIP assays were performed using genomic DNA isolated from liver fragments of human and rat or dog spleen, and the binding of ERRγ to the endogenous promoters of miR-433 and of miR-127 of each species was detected using specific ERRγ antibodies.

We next determined the physical association of ERRγ with the endogenous miR-433 and miR-127 promoters using ChIP assays. The miR-433/127 loci cloned from human, rat, or dog was overexpressed in mouse Hepa-1 cells by transfection of an expression plasmid containing the loci of each species (Figure S2, see below description), respectively. The binding of ERRγ to the promoter of miR-433 and of miR-127 of each species was detected using specific ERRγ antibodies and corresponding species-specific primers. As shown in Figure 5, ERRγ was found to Co-IP on both the miR-433 and miR-127 promoters of human, rat, or dog. Because specific ChIP primers for each species were used and the miR-433/127 loci of each species was transfected into the cells of a different species (mouse), it is postulated that the ChIP assays would detect ERRγ binding to the miR-433 and miR-127 promoters of each species. To further confirm this result, the direct association of ERRγ with miR-433 and miR-127 promoters of each species *in vivo* was assessed using ChIP assays. ERRγ was co-immunoprecipitated on the ERRE containing the endogenous promoter regions of miR-433 and miR-127 in the liver of human and rat, and dog spleen, respectively (Figure 5b).

### Artificial Expression of miR-433 and miR-127 *In Vitro* in Cells

**Figure 6:**
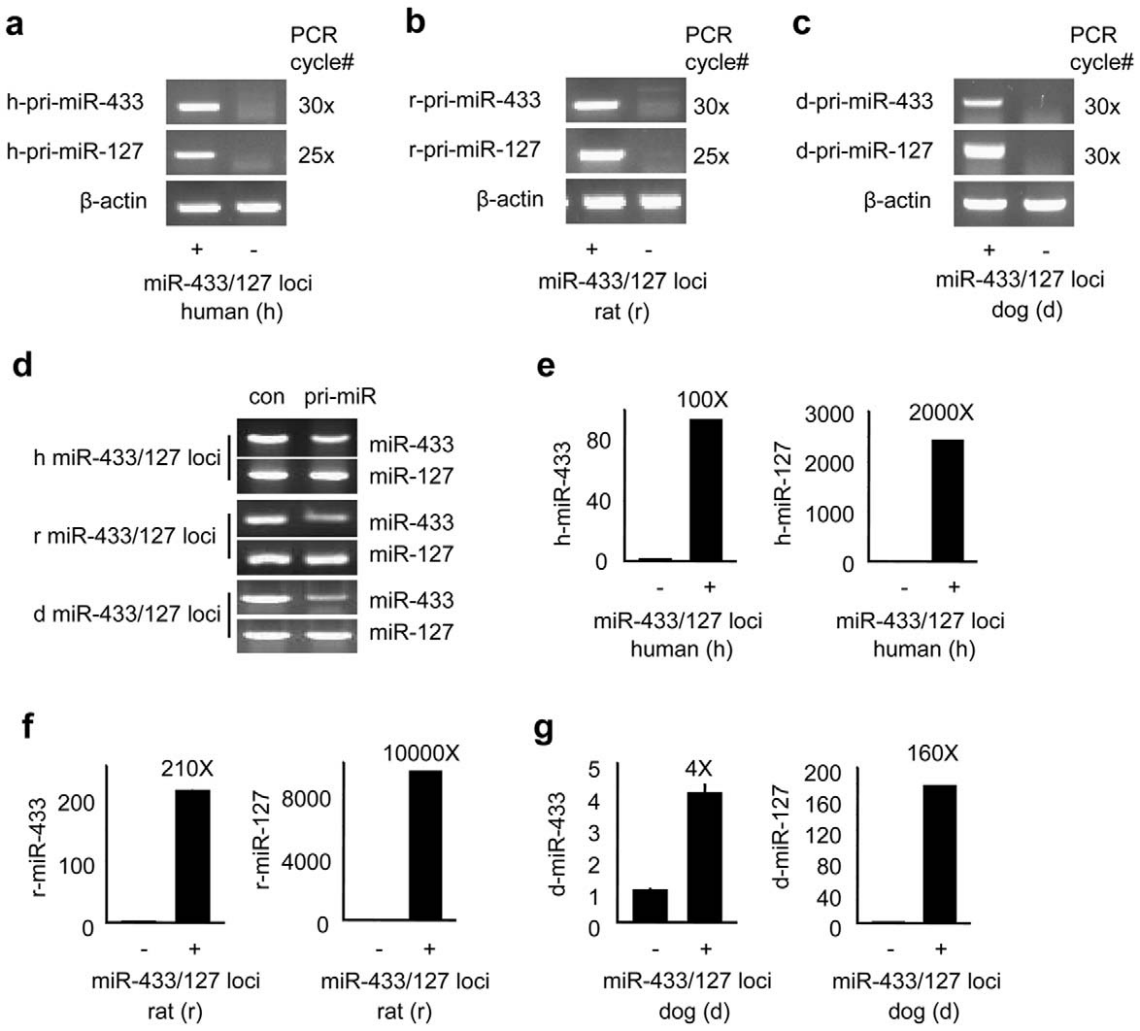
Transcriptional expression of miR-433 and miR-127 from the miR-433/127 loci in human, rat, and dog. (Panels **a–c**) Semi-quantitative RT-PCR analysis of pri-miR-433 and pri-miR-127 expression transcribed from the human (**a**), rat (**b**), and dog (**c**) miR-433/127 loci expression plasmid. PCR cycles used are indicated in each panel. (**d**) Semi-quantitative RT-PCR analysis of pri-miR-433 and pri-miR-127 expression transcribed from the human, rat, or dog miR-433/127 loci expression plasmid in Hepa-1 cells. con: each loci expression plasmid was used as a control. (Panels **e–g**) Real-time PCR analysis of the expression of mature miR-433 and miR-127 transcribed from the recombinant human (**e**), rat (**f**), and dog (**g**) miR-433/127 loci expression plasmid. Total RNA containing miRNA was isolated and used for semi-quantitative (**a–d**) and real-time (**e–g**) PCR analysis. Primers used to determine the expression of primary transcripts of miR-433 and of miR-127 are located surrounding the precursors of miR-433 and of miR-127. Data in **e-g** are represented as mean ± SE.

Our previous studies showed that the mouse miR-433 and miR-127 genes overlapped and the expression of mouse miR-433 and miR-127 was controlled by independent promoters [3], [4]. To determine if miR-433 and miR-127 in human, rat, and dog can be independently and differentially transcribed using each miRNA's own promoter, we cloned a large (∼4.5 kb) human, rat, or dog genomic DNA fragment containing miR-433 and miR-127 and their promoter regions into pMIR-REPORT vector (Figure S2). The pMIR-REPORT vector was modified in which the original promoter of the vector and the coding region of luciferase gene were deleted. This would allow us to determine if the transcriptional expression of miR-433 or miR-127 could be driven by its own promoter from the endogenous miR-433/127 loci. The recombinant human, rat, or dog miR-433/127 loci expression vector (designated pMIR-REPORT-NoMp-human, rat or dog) was then transfected into Hepa-1 cells and the expression of miR-433 and miR-127 primary transcripts was examined using semi-quantitative RT-PCR. Potential PCR amplification from genomic DNA contamination was eliminated by treating the total RNA with DNase I. As shown in Figures 6a–c, primary transcripts of the human (a), rat (b), or dog (c) miR-433 and miR-127 were easily detected in cells that over-expressed the recombinant expression vector, respectively. In addition, the expression level of pri-miR-433 was markedly lower than that of pri-miR-127 (Figure 6d) in all three species, suggesting pri-miR-433 and pri-miR-127 were transcribed differentially from an independent transcription unit.

We next determined expression levels of the mature miR-433 and miR-127 in Hepa-1 cells after recombinant vector over-expression. Compared with non-vector transfected cells, the human miR-433 was ∼100 fold over-expressed whereas the human miR-127 was ∼2000 fold over-expressed (Figure 6e). Similarly, the rat miR-433 and miR-127 was ∼210 fold and ∼10000 fold ovexpressed (Figure 6f) and the dog miR-433 and miR-127 was ∼4 fold and ∼160 fold ovexpressed (Figure 6g), respectively. Thus, the expression of human miR-127 was ∼20 times higher than human miR-433, the expression of rat miR-127 was ∼47 times higher than rat miR-433, and the expression of dog miR-127 was ∼40 times higher than dog miR-433. These differential expression results provided further evidence that miR-433 and miR-127 produced from the miR-433-127 loci were transcribed from two separate promoters in human, rat, and dog.

### Down-Regulation of the Imprinted Gene Rtl1 by Overexpression of miR-127

**Figure 7:**
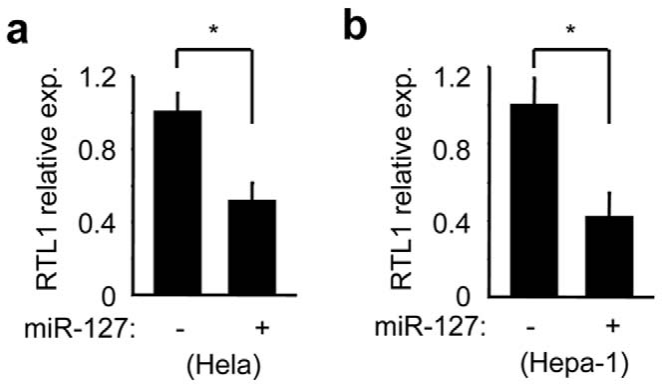
Strand-specific real-time PCR analysis of Rtl1 expression. The expression vector of miR-127 was transfected in human Hela and mouse Hepa-1 cells and the expression of Rtl1 was determined by a modified strand-specific q-PCR. Data is represented as mean ± SE (*p<0.01).

Because miR-127 is located in an imprinted region encoding Rtl1, we determined if changes in miR-127 expression would affect the expression of Rtl1. We overexpressed miR-127 in human Hela and mouse Hepa-1 cells using an expression vector of miR-127 and the level of Rtl1 was assessed using a strand specific q-PCR analysis. Interestingly, ectopic expression of miR-127 resulted in a significant reduction in Rtl1 expression in both cells (Figure 7). This data suggests that miR-127 may function as a siRNA to down-regulate its host gene expression.

## Discussion

Although overlapping genes occur frequently in viral and prokaryotic genomes as well as organelles such as mitochondria [6], it was previously believed that they occurred less frequently in eukaryotic nuclear genomes. Although earlier studies reported overlapping genes in the genome of humans and other species [7]–[13], recent reports showed a more frequent occurrence of overlapping genes in human and other mammalian genomes [14]–[16]. In spite of the growing evidence for the protein coding overlapping genes in mammalian species, little is known about the origin, evolution, or cross-species conservation of the non-protein coding overlapping miRNA genes.

Our results presented in this study provide evidence that the miR-433 and miR-127 overlapping genes have a higher rate of conservation in mammalian species. This is in agreement with the notion that the rate of evolution is expected to be slower in overlapping genes [17]. Lipman proposed that the higher rate of conservation of noncoding sequences of some genes is explained by the presence of antisense transcripts [18]. Our published results showed that miR-433 and miR-127 genes are overlapped in a 5′–3′ unidirectional way in mouse [3] and these two non-coding genes have an antisense transcript, RTL1 [19]. Because the viral and prokaryotic genomes were under strong evolutionary pressure to minimize genome size, overlapping genes represent an excellent strategy to condense a maximum amount of information into short nucleotide sequences [20].

Why is the miR-433 and miR-127 overlapping gene structure in mammalian species so conserved? One possibility would be that mutations in the overlapping region may result in expressional and functional changes of both genes. For instance, if mutations occurred in the region between pre-miR-433 and pre-miR-127, it would be likely to affect both the processing of mature miR-433 and the regulation of miR-127 expression. On the other hand, we observed a common transcriptional mechanism governing the expression of miR-433 and miR-127 in mammals, which involved ERR family members and orphan receptor SHP. The conservation of the transcriptional regulation of miR-433 and miR-127 further supports the notion that the miR-433/127 loci in mammals might be evolved from an ancient common origin.

Genomic imprinting refers to an epigenetic phenomenon by which certain genes (e.g. imprinted genes) are expressed in a parent-of-origin specific manner. The grouping of imprinted genes within clusters allows them to share common regulatory elements, such as non-coding RNAs and differentially methylated regions (DMRs). It has been reported that the miR-433/127 locus is located in the imprinted region Dlk1/Gtl2 and transcribed in an antisense orientation to a retrotransposon-like gene Rtl1 [21]–[24]. A recent study showed that deletion of Gtl2 and the imprinted non-coding RNA in mice induced lethal parent-origin-dependent defects [25]. The imprinted expression of miR-127 has been shown to undergo DNA methylation regulation in mouse embryos [26] and cancer cells [27]. These results suggest that the expression of miR-433/127 is not only under transcriptional control as elucidated in our previous [3], [4] and the present study, but also subjects to epigenetic regulation. Alteration of those miRNAs expression may in turn affect the expression of the imprinted genes, as evidenced by the down-regulation of Rtl1 by miR-127 in this study.

In conclusion, our studies for the first time provide evidence for a conserved structure and transcriptional regulation of the clustered miR-433 and miR-127 genes in mammals, including humans. Given the fact that many miRNAs are clustered on different chromosomes, we propose that overlapping gene structure and conserved regulated expression may represent a common feature for other genomically clustered miRNAs.

## Methods

### Bioinformatics Analysis

The precursor sequences of miR-433 and miR-127 were downloaded from the Sanger Institute (http://microrna.sanger.ac.uk/sequences/). BLASTN search of genome sequences of different species was completed online and a 5 kb genomic sequence surrounding the miR-433 and miR-127 precursors in each species was extracted manually. Multiple sequence alignment was done using ClustalW. MatInspector of Genomatix Software Suite was used to predict the transcription factor binding sites in the promoter regions of miR-433 and miR-127 in different species, which was completely using Default parameters (http://www.genomatix.de/products/MatInspector/index.html).

### Long PCR

Genomic DNAs were extracted from human, rat, and dog liver specimens using DNasesy Kit (Qiagen). The specimens were purchased from commercial resources. Long PCR was completed using TaKaRa *LA Taq*™ DNA Polymerase according to the manufacturer's instructions (TaKaRa Takara Bio Madison, WI). All primer sequences are provided in the Supplementary Material Table S1.

### Expression Vector Construction

In order to take the advantage of restriction sites of Asc I and Pac I, a random 300 bp DNA fragment was cloned into the BamH I site of pNEB193 (New England Biolabs) to generate pNEB193-insert. pMIR-REPORT Luciferase expression vector (Ambion) was digested using EcoR I and Hind III to delete the promoter of vector and the coding region of luciferase gene to produce pMIR-REPORT vector without mammalian expression promoter, which was designated as pMIR-REPORT-NoMp. Then the pNEB-insert was cut using EcoR I and Hind III and the produced fragment was inserted into the EcoR I and Hind III sites of pMIR-REPORT-NoMp, which produced the pMIR-REPORT-Insert. pMIR-REPORT-NoMp-Insert was digested using Asc I and Pac I. As a result, the insert on the pMIR-REPORT-NoMp-Insert was deleted and the produced vector is pMIR-REPORT-NoMp with Asc I and Pac I site. Finally, the long DNA fragments containing human, rat or dog miR-433 and miR-127 loci were inserted into Asc I and Pac I sites of pMIR-REPORT-NoMp. Cloning procedures and construct maps are provided in the Supplementary Material Figure S2.

### Transient Transfection

The promoters of miR-433 and miR-127 from human, rat and dog were cloned into a pGL3 basic vector, respectively. Hela cells were maintained in Dulbecco's Modified Eagle's Medium in the presence of 10% fetal bovine serum. All constructs were verified by sequencing. Expression vectors of SHP, ERRγ, ERRα, and ERRβ were available in our laboratory. For luciferase assays, cells were plated in 24-well plates one day before transfection and transfection was carried out using Fugene HD (Roche). Total DNA in each transfection was adjusted by adding appropriate amounts of pcDNA3 empty vector. Approximately 48 hr post-transfection, cells were harvested and luciferase activities were measured and normalized against β-galactosidase activities as an internal control. The transfection experiments were carried out independently 3 times with similar efficiency and one representative result is shown.

### Chromatin Immunoprecipitation (ChIP) Assays

ChIP Assays were performed using the ChIP Assay Kit (Upstate Biotechnology, Lake Placid, NY). Hepa-1 cells were cultured until 70%–80% confluence. Five µg of pMIR-REPORT-NoMp vector containing miR-433/127 loci of human, rat or dog, respectively, was transfected into Hepa-1 cell on 60 mm culture plate. The cells were used for ChIP assays 48 hr post-transfection. Chromatin was cross-linked with 1% formaldehyde at 37**°**C for 10 min. Cells were washed with cold PBS twice and disrupted in SDS Lysis Buffer containing the protein inhibitor cocktail. Chromatin was sonicated to shear DNA to an average length between 200 bp and 1000 bp as verified by agarose gel. The sonicated cell supernatants were diluted 10-fold in ChIP Dilution Buffer containing the protein inhibitor cocktail and an aliquot of the solution was reserved for input control. Ten microgram of ERRγ antibodies (Aviva Systems Biology, San Diego, CA) was added and the chromatin solution was gently rotated overnight on ice. The protein A agarose slurry was added to the antibody-bound chromatin solution and incubated at 4°C for 1 hr with constant rotation. The agarose beads were collected by centrifugation, washed and the antibody-bound chromatin was released from the agarose beads. Finally, the DNA was purified by phenol/chloroform extraction and ethanol precipitation. The binding region was detected in PCR reactions. A 10 kb region downstream from the binding site was used as a negative control. All primer sequences are provided in the Supplementary Material Table S2.

For *in vivo* ChIP assays, 100 mg of frozen liver of human and rat or dog spleen were homogenized in 1 ml PBS with 1 x protease cocktail (Sigma) and fixed in 10% formaldehyde in PBS for 10 min, then the protocol from Upstate Biotechnology was followed (Cat# 17–295). Ten µg of ERRγ antibodies was used to incubate with the liver lysate overnight. The final extracted DNA was resuspended in 20 µl H_2_O and 1 µl was used as template for amplification of each gene promoter using ChIP assays primers listed in the Supplementary Material Table S2.

### RT-PCR Quantification of miRNAs

zFive µg of recombinant plasmid of miR-433/127 loci of human, rat and dog was transfected into Hepa-1 cells, respectively. Cells were harvested 48 hr post-transfection and total RNA containing microRNA was extracted. Genomic DNA and plasmid DNA contamination in the total RNA was removed by DNase I digestion. Two µg of total RNA without DNA contamination was reverse transcribed into cDNA. Real-time reverse transcriptase polymerase chain reaction (RT-PCR) quantification of miRNA expression was carried out using TaqMan® MicroRNA Assays Kit (Applied Biosystems Inc. Foster City CA) according to the manufacturer's instructions. Briefly, cDNAs were synthesized from total RNA using gene-specific RT primers. 15 **µ**l of Reverse transcription reactions contained 10 ng RNA samples, 3 **µ**l of RT primer, 0.15 **µ**l of 100 mM dNTP, 0.19 **µ**l of RNA inhibitor, 1.5 **µ**l of RT buffer and 1 **µ**l of MultiScribe Reverse Transcriptase. The 15 **µ**l reaction were incubated for 30 minutes at 16°C, 30 min at 42°C, 5 min at 85°C, then held at 4°C. Real-time PCR was performed using an Applied Biosystems 7900 Sequence Detection system. The 15 **µ**l PCR reaction included 1 **µ**l RT product, 7.5 **µ**l of Taqman 2 X universal PCR master Mix no AmpErase UNG (Applied Biosystems, Foster City CA) and 0.75 **µ**l of 20 x Taqman MicroRNA assay. Reactions were incubated in a 96-well optical plate at 95°C for 10 minutes, followed by 40 cycles of 95°C for 15 seconds and 60°C for 1 min. The threshold cycle (Ct) was determined using default threshold settings. The Ct value is defined as the fractional cycle number at which the fluorescence passes the fixed threshold. All experiments were done in triplicates and repeated three times. snoRNA202 was used as an internal control to normalize RNA input in the real-time RT-PCR assay. Statistical analyses were performed by the 2^−ΔΔCT^ method [4]. Data were then analyzed using the equation 2^−ΔΔCT^, where ΔΔ CT = (CT, Target -CT, snoRNA202)Target sample- (CT, Target-CT, snoRNA202) Calculator. The expression level of primary transcript of miR-433 and miR-127 was determined by semi-quantitative PCR using primers listed in Supplementary Material Table S3. Due to high expression of primary transcript of miR-433 and mIR-127, different PCR cycles were used (see Figure 6).

The slope of the standard curve can be used to determine the exponential amplification efficiency of the PCR reaction by the following formula: Efficiency = 10^(−1/slope)^−1. In this study, we calculated the amplification efficiency of miR-433 and miR-127 by drawing the standard curve. We found that miR-433 and miR-127 had almost identical PCR amplification efficiency (Supplementary Material Table S4). Based on this information, we made a direct comparison between the expression level of miR-433 and miR-127.

### Semi-Quantitative RT-PCR for miR-433 and miR-127 Primary Transcripts

In order to avoid the effect of endogenous miR-127 and miR-433 primary transcripts in Hela cells, we overexpressed the pMIR-Report human433/127 loci, pMIR-Report rat433/127 loci, and pMIR-Report dog433/127 loci in Hepa1 cells. Twenty-four hours after transfection, we collected cells and isolated total RNA using the miRNAease Kit (Qiagen). The total RNA was transcribed into cDNA using Superscript III. It is possible that different primer pairs may have different amplification efficiency. In this study, we used pMIR-Report-human 433/127, pMIR-Report-rat433/127, pMIR-Report-dog433/127 plasmids as the standard template to determine the PCR efficiency, then compared the expression level of miR-433 and miR-127 primary transcripts.

### Strand-Specific Real-Time RT-PCR

We used a simple modification of the RT-PCR reaction that allows the sense and antisense mRNAs to be distinguished without having to truncate the cDNA which requires only one primer set. Instead of using Oligo(dT) or random hexamers to prime first-strand cDNA synthesis, Rtl1 specific RT primers were used for reverse transcription. Rtl1 specific primers will bind only to the Rtl1 mRNA, and miR-433 and miR-127 primary transcripts will not be transcribed into cDNA. The first-strand reactions then served as targets for real time PCR. The mouse cell line Hepa1 and human cell line Hela were transfected with miR-127 expression vector. Twenty-four hrs post-transfection, total RNA was prepared from the transfected cells using the miRNAeasy Kit (Qiagen). Total RNA was treated with DNase I (Ambion) to remove contaminated genomic DNA. The reverse transcription was completed using Superscript III Rnase H-reverse transcriptase (Invitrogen). The manufacturer's protocol was followed, except that 50 pmol of Rtl1 specific RT primer instead of Oligo(dT) or random hexamers, was annealed to the RNA. The expression level of Rtl1 was determined using SyberGreen real-time PCR.

### Statistical Analysis

All the experiments were repeated at least three times and the error bars represent the standard error of the mean (SE). Statistical analyses were carried out using Student's unpaired t test; p<0.01 was considered statistically significant.

## Supporting Information

Figure S1 Promoter analysis of miR-433 and miR-127 luciferase reporters of human, rat and dog. Transient transfection assays to determine ERRα and ERRβ regulation of miR-433 and miR-127 promoter (pro.) transactivation of human, rat, and dog, respectively. The promoters of pri-miR-433 and pri-miR-127 were cloned into a pGL3-basic vector, respectively. Hela cells were transfected with the miR-433Luc or miR-127Luc in the presence of ERRα and ERRβ expression plasmids. Luciferase (luc.) activities (act.) were determined, which were normalized by β-gal activities. Data are represented as mean ± SE.
(1.36 MB TIF)

Figure S2 Procedures for cloning the miR-433/127 loci expression vector of human, rat, or dog.
(4.03 MB TIF)

Table S1 Primers used to generate miR-433/127 loci expression vector.
(4.02 MB TIF)

Table S2 Primers used for ChIP assays.
(4.02 MB TIF)

Table S3 Primers used for semi-quantitative PCR analysis.
(4.02 MB TIF)

Table S4 Q-PCR amplification efficiency using primers specific for miR-433 and miR-127.
(3.00 MB TIF)
