# Supplementary figures and images for: A Conserved Gene Structure and Expression Regulation of miR-433 and miR-127 in Mammals
Source: PLoS One. 2009 Nov 25;4(11):e7829. doi: 10.1371/journal.pone.0007829 (PMC2778354; doi:10.1371/journal.pone.0007829)

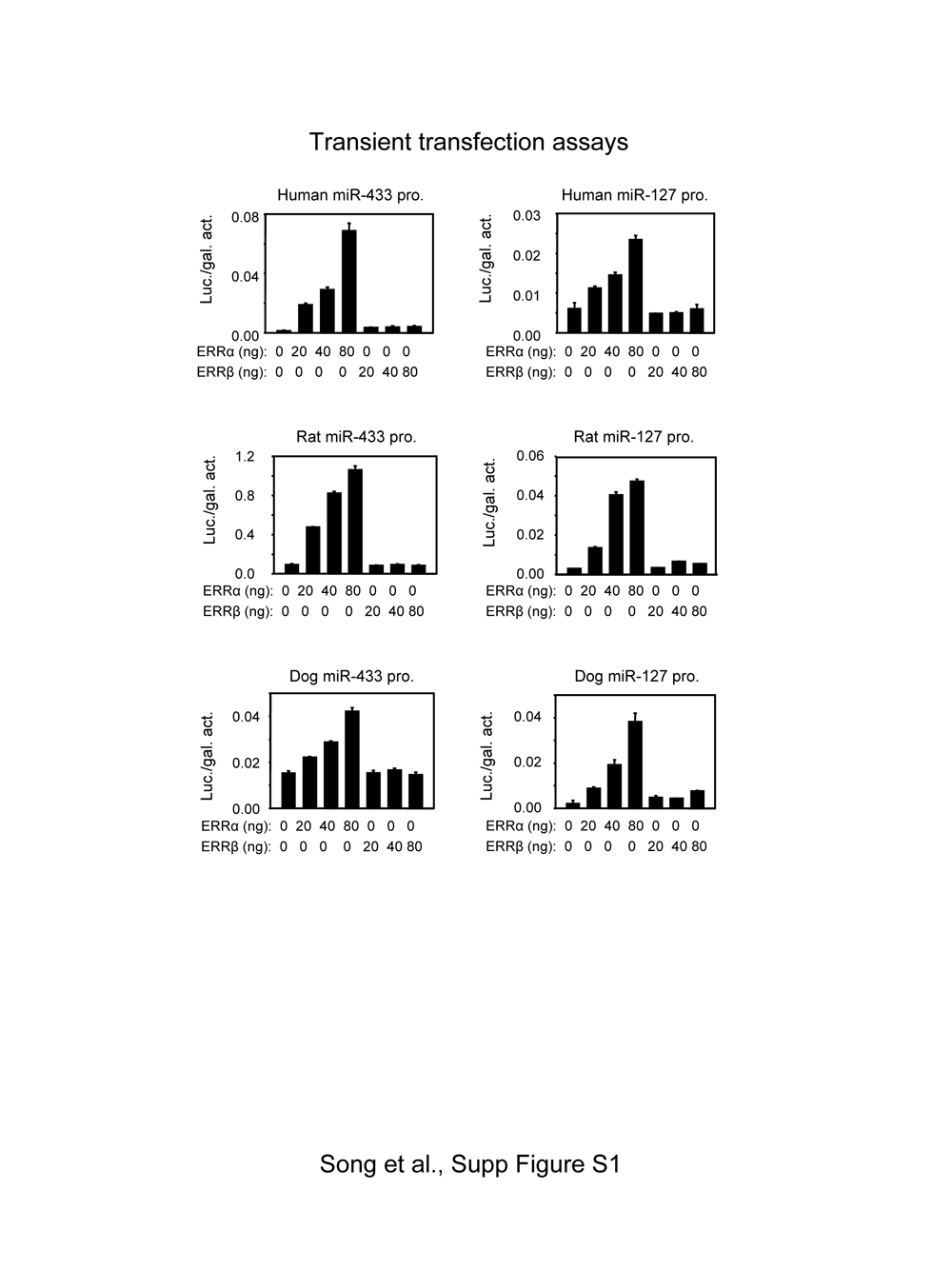

Supplement: Figure S1 — Promoter analysis of miR-433 and miR-127 luciferase reporters of human, rat and dog. Transient transfection assays to determine ERRα and ERRβ regulation of miR-433 and miR-127 promoter (pro.) transactivation of human, rat, and dog, respectively. The promoters of pri-miR-433 and pri-miR-127 were cloned into a pGL3-basic vector, respectively. Hela cells were transfected with the miR-433Luc or miR-127Luc in the presence of ERRα and ERRβ expression plasmids. Luciferase (luc.) activities (act.) were determined, which were normalized by β-gal activities. Data are represented as mean ± SE. (1.36 MB TIF) [file pone.0007829.s001.tif]

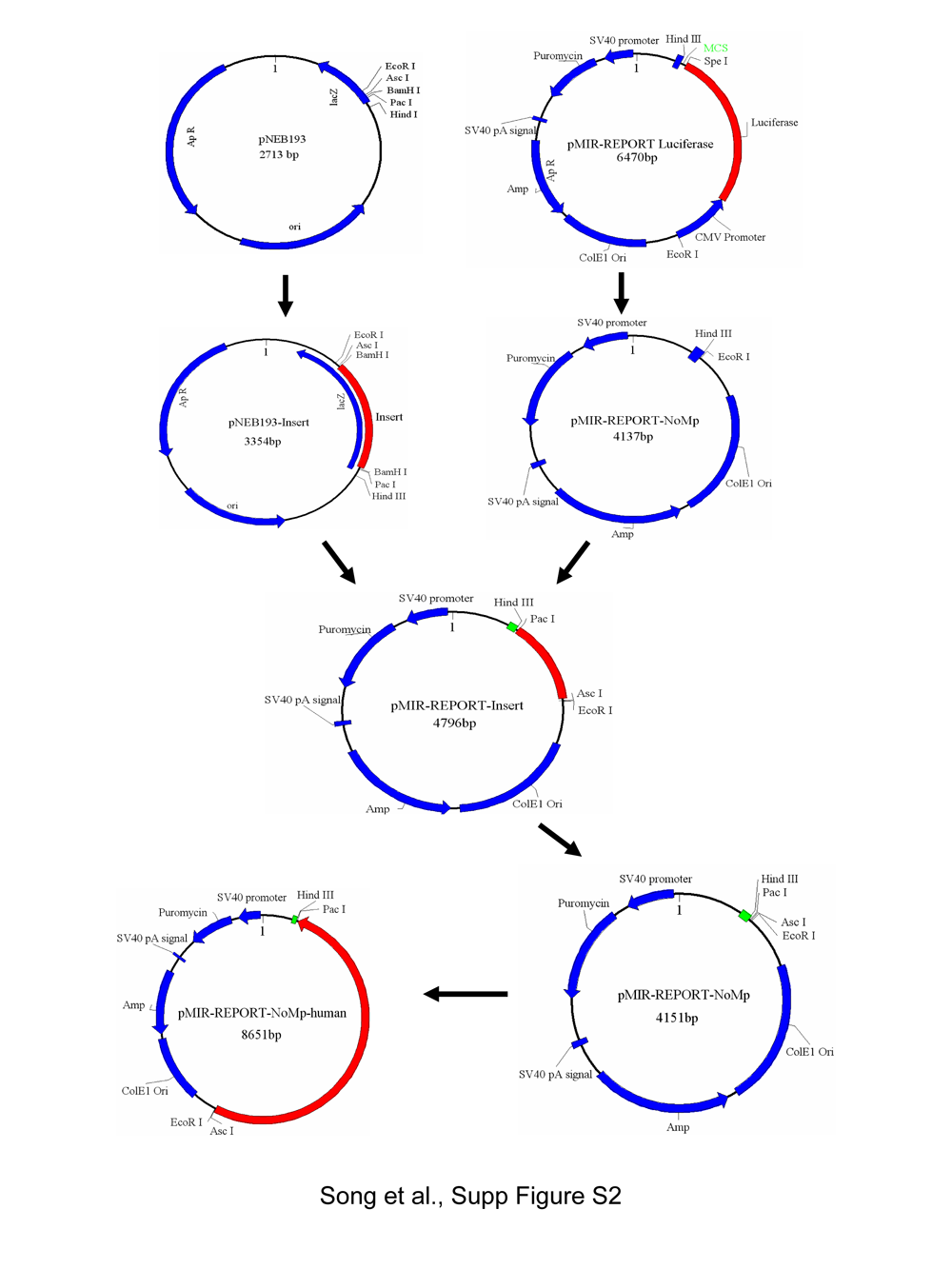

Supplement: Figure S2 — Procedures for cloning the miR-433/127 loci expression vector of human, rat, or dog. (4.03 MB TIF) [file pone.0007829.s002.tif]

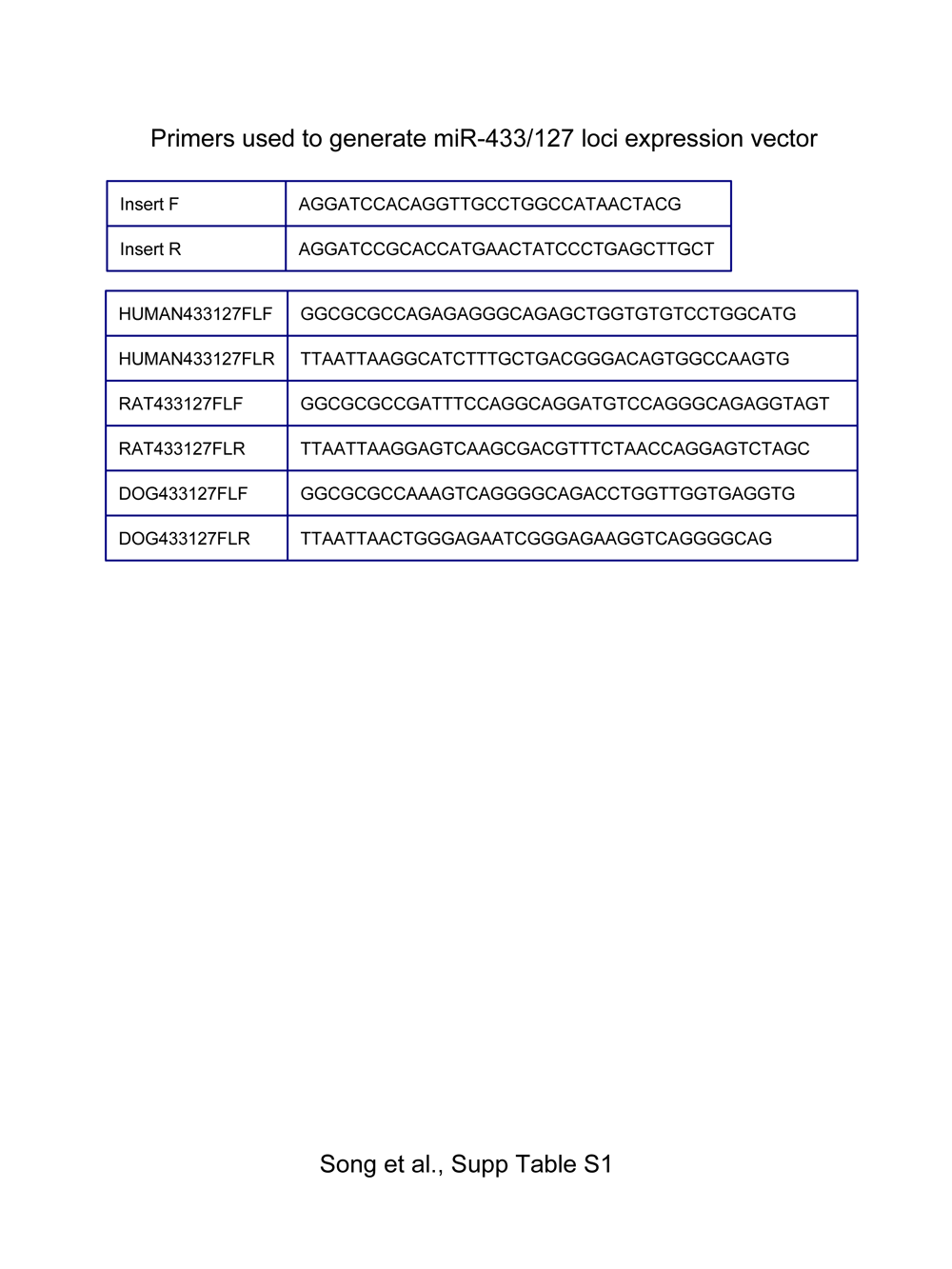

Supplement: Table S1 — Primers used to generate miR-433/127 loci expression vector. (4.02 MB TIF) [file pone.0007829.s003.tif]

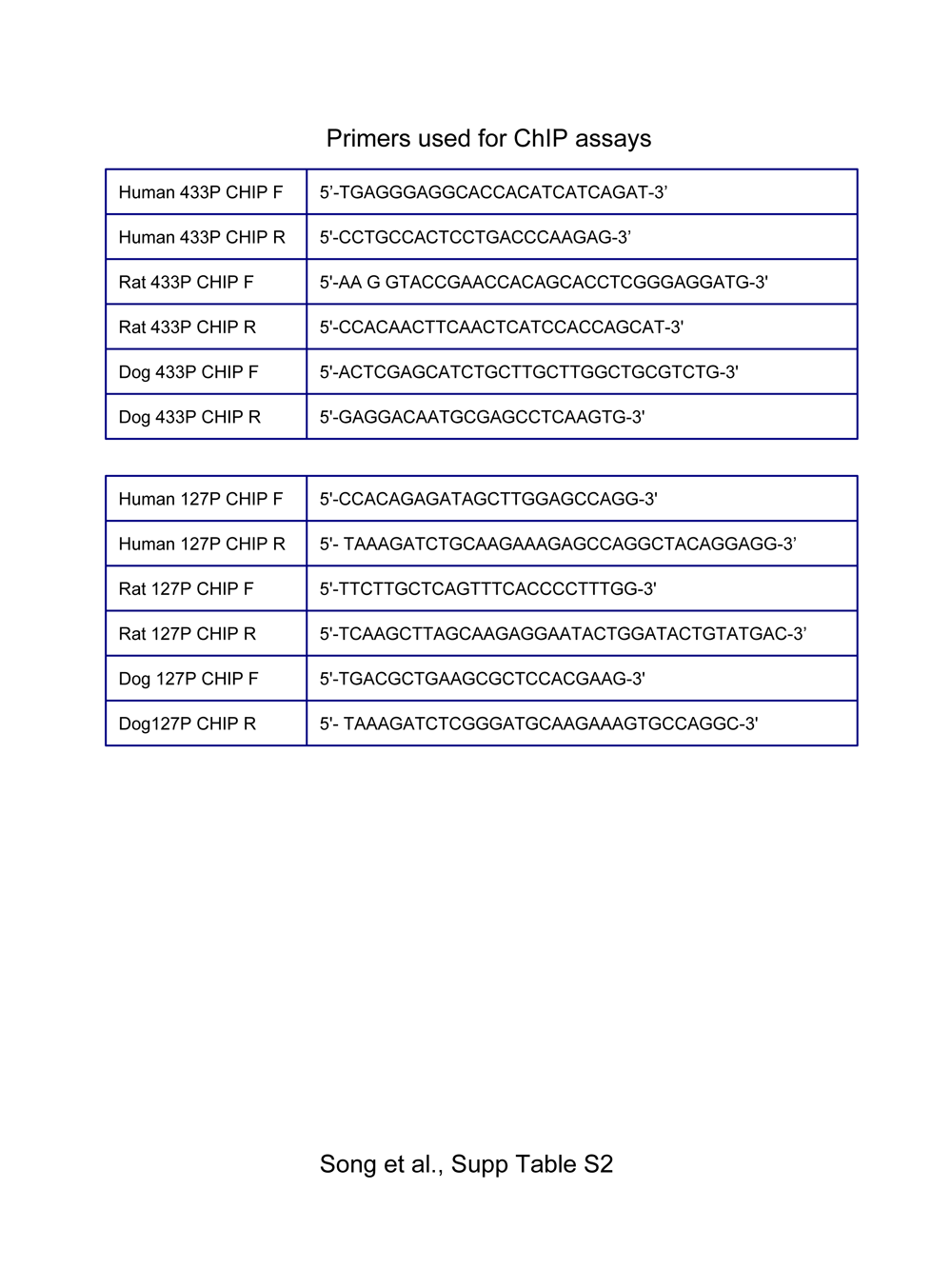

Supplement: Table S2 — Primers used for ChIP assays. (4.02 MB TIF) [file pone.0007829.s004.tif]

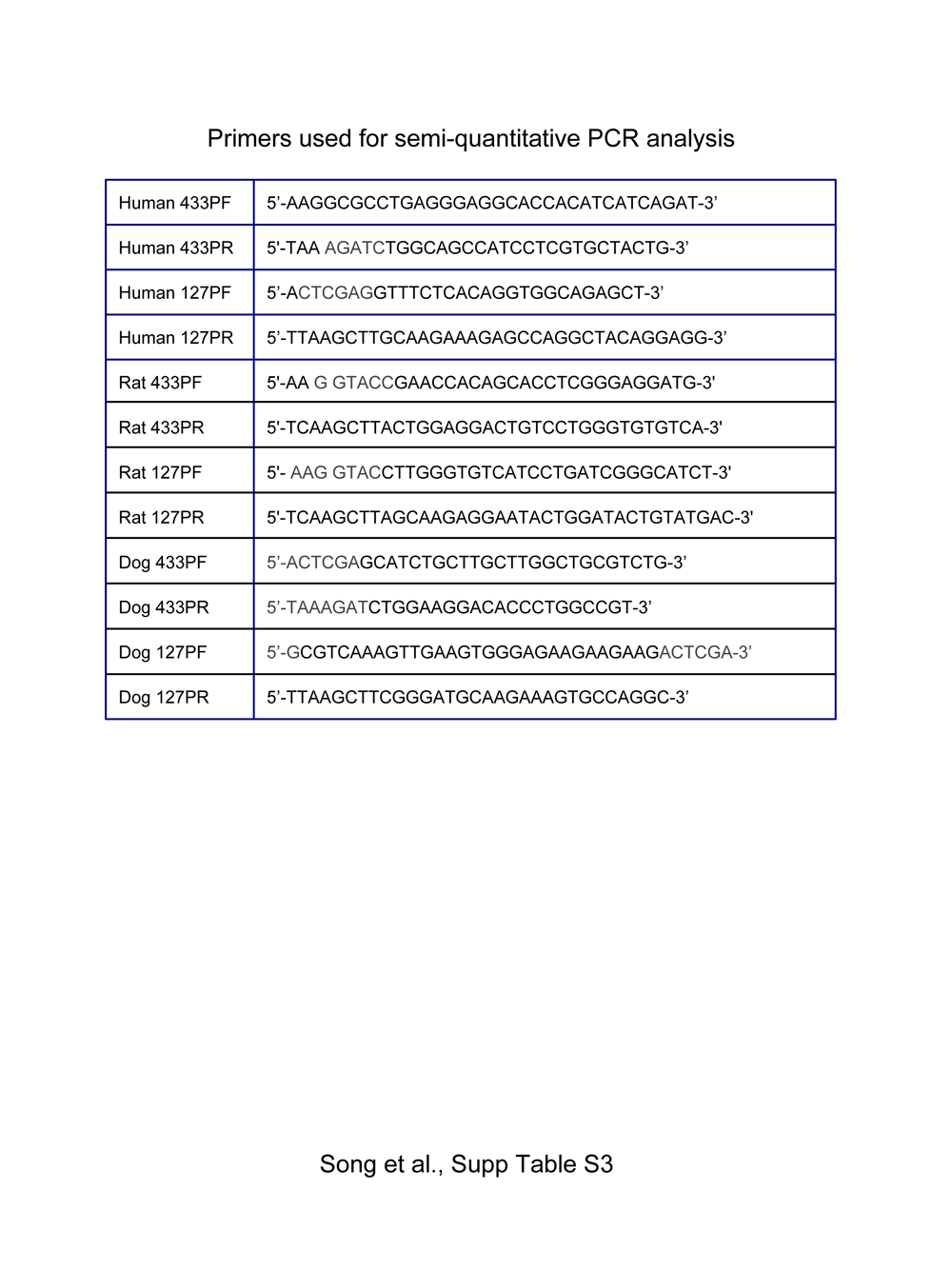

Supplement: Table S3 — Primers used for semi-quantitative PCR analysis. (4.02 MB TIF) [file pone.0007829.s005.tif]

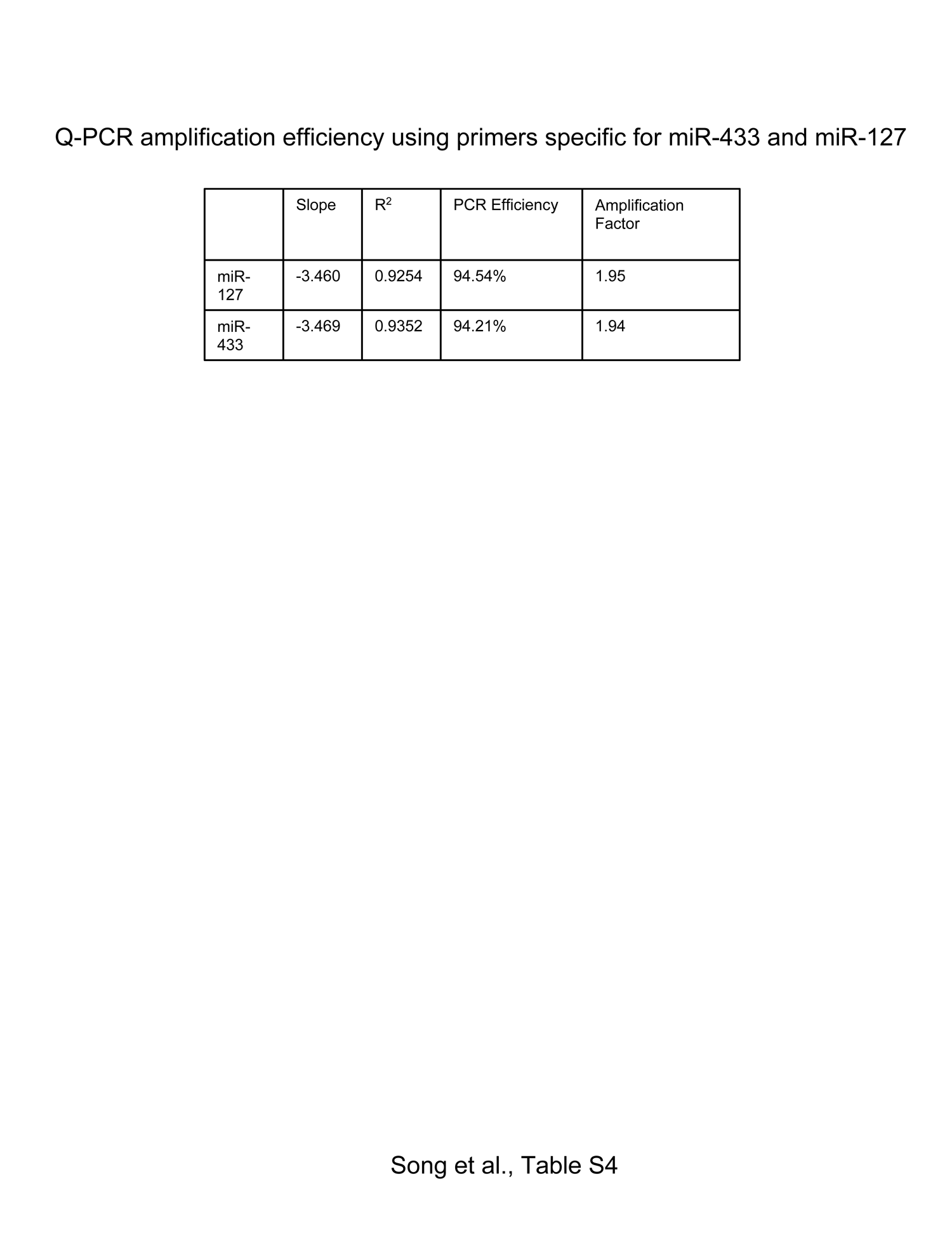

Supplement: Table S4 — Q-PCR amplification efficiency using primers specific for miR-433 and miR-127. (3.00 MB TIF) [file pone.0007829.s006.tif]
